# Genomic dissection of maternal, additive and non-additive genetic effects for growth and carcass traits in Nile tilapia

**DOI:** 10.1186/s12711-019-0522-2

**Published:** 2020-01-15

**Authors:** Rajesh Joshi, Theo H. E. Meuwissen, John A. Woolliams, Hans M. Gjøen

**Affiliations:** 1 Department of Animal and Aquacultural Sciences, Norwegian University of Life Sciences, P.O. Box 5003, 1432 Ås, Norway; 2 The Roslin Institute, Royal (Dick) School of Veterinary Studies, University of Edinburgh, Easter Bush, Midlothian, EH25 9RG UK

## Abstract

**Background:**

The availability of both pedigree and genomic sources of information for animal breeding and genetics has created new challenges in understanding how they can be best used and interpreted. This study estimated genetic variance components based on genomic information and compared these to the variance components estimated from pedigree alone in a population generated to estimate non-additive genetic variance. Furthermore, the study examined the impact of the assumptions of Hardy–Weinberg equilibrium (HWE) on estimates of genetic variance components. For the first time, the magnitude of inbreeding depression for important commercial traits in Nile tilapia was estimated by using genomic data.

**Results:**

The study estimated the non-additive genetic variance in a Nile tilapia population of full-sib families and, when present, it was almost entirely represented by additive-by-additive epistatic variance, although in pedigree studies this non-additive variance is commonly assumed to arise from dominance. For body depth (BD) and body weight at harvest (BWH), the proportion of additive-by-additive epistatic to phenotypic variance was estimated to be 0.15 and 0.17 using genomic data (P < 0.05). In addition, with genomic data, the maternal variance (P < 0.05) for BD, BWH, body length (BL) and fillet weight (FW) explained approximately 10% of the phenotypic variances, which was comparable to pedigree-based estimates. The study also showed the detrimental effects of inbreeding on commercial traits of tilapia, which was estimated to reduce trait values by 1.1, 0.9, 0.4 and 0.3% per 1% increase in the individual homozygosity for FW, BWH, BD and BL, respectively. The presence of inbreeding depression but lack of dominance variance was consistent with an infinitesimal dominance model for the traits.

**Conclusions:**

The benefit of including non-additive genetic effects for genetic evaluations in tilapia breeding schemes is not evident from these findings, but the observed inbreeding depression points to a role for reciprocal recurrent selection. Commercially, this conclusion will depend on the scheme’s operational costs and resources. The creation of maternal lines in Tilapia breeding schemes may be a possibility if the variation associated with maternal effects is heritable.

## Background

This work is a part of a wider study on the non-additive genetic effects in Nile tilapia and their potential use in tilapia breeding programs. A previous study [1] used the classical approach to partition the phenotypic variance observed in a diallel mating design into additive, non-additive and maternal genetic components using pedigree information to generate the additive and dominance relationship matrices. These variance components were inferred from the variances within and between full-sib families, with the latter further decomposed into variances among sires and among dams.

Pedigree-based selection methods have been gradually supplemented with genomic information in various livestock species [2], and in some commercial aquaculture species [3]. Based on the expectation that genomics will lead to improved accuracy of breeding values and provide more detailed information [4], there has been a growing interest in quantifying and using non-additive genetic sources of phenotypic variation to achieve breeding objectives more effectively. The development of genomic technologies has raised new challenges for a complete understanding of the interpretation of analyses based on genomic information, and to what degree they are equivalent to classical decompositions of variance based on pedigree. The availability of genomic information in Nile tilapia [5] has offered the opportunity to respond to these challenges in an important aquaculture species. Therefore, the first aim was to compare estimates of the genetic variance components obtained when using relationship matrices derived from pedigree or from genomic information in a study designed to estimate non-additive variances.

The genomic best linear unbiased prediction (GBLUP) model for a trait builds a matrix of relationships between all individuals based on genomic data, and uses it to partition the phenotypic variance into components, and hence to predict breeding values. Assumptions made in methods for constructing the relationship matrix have a direct effect on the accuracy of the results. There are a variety of methods for constructing relationship matrices using genomics, which typically differ in the scaling parameters [6–8] used, and which make it difficult to compare the variance components and heritabilities obtained from them. One method of comparison was published by Legarra [9], who showed that it is necessary to re-scale the relationship matrices to the same reference population. The construction of relationship matrices often assumes the state of Hardy–Weinberg equilibrium (HWE), e.g. in the VanRaden matrices [7] as used by the GCTA software [10], and the state of linkage disequilibrium (LD) in the population [11]. These assumptions influence the orthogonality of the estimates of the components of the genetic variance (e.g. additive, dominance, etc.) and, hence, whether the estimates change when other components are included in the model. Changes in estimates arising from non-orthogonality also compromise the validity and generality of their biological interpretation [12, 13]. Thus, the second aim of this study was to examine the impact of assumptions about HWE on the relationship matrices and their impact on the estimates of variance components.

Inbreeding depression is a natural phenomenon that is widely assumed to be deleterious for traits of commercial importance and, thus, can have major practical implications [14–17]. Its impact is greater in populations with a small effective population size (${\text{N}}_{\text{e}}$) compared to those with a large ${\text{N}}_{\text{e}}$, due to more efficient purging of deleterious alleles in the latter [18, 19]. Thus, inbreeding depression is a concern to breeders since ${\text{N}}_{\text{e}}$ is often restricted in breeding populations. The use of genomic data allows a direct assessment of the impact of homozygosity from its variation among individuals, rather than relying on changes in homozygosity predicted from pedigree-based inbreeding. To the best of our knowledge, estimates of inbreeding depression in Nile tilapia are not available, even based on pedigree data. Thus, the final aim of this paper was to quantify the effect of inbreeding depression for important commercial traits in Nile tilapia using genomic data.

On a wider scale, our aim was to assess the impact of genomic methods on dissecting maternal, additive and non-additive genetic variances in important commercial traits of Nile tilapia. The insights gained into their genetic architecture will provide important information for the future design and operation of breeding programs in an important commercial species.

## Methods

### Experimental design, phenotypes and genotypes

The population used in this study and the experimental design were previously described [1]. In short, the population was obtained from the reciprocal crossing of two parent groups, A and B, of Nile tilapia. Matings were partially factorial, such that each parent, male or female, had offspring that were both full- and half-sibs. All offspring were subjected to a hormonal treatment, which is a normal aquaculture procedure, to avoid sexual maturation that interrupts growth, especially among females; i.e. offspring were either males or sex-reversed males. Offspring were reared in three batches, harvested over 8 days following 6 to 7 months in the grow-out ponds, and filleted by three filleters. Body weight at harvest (BWH), body depth (BD), body length (BL), body thickness (BT), fillet weight (FW) and fillet yield (FY) were recorded. These phenotypes were obtained on 2524 individuals, with 1318 and 1206 from each of the two reciprocal crosses, in 155 full-sib families.

Of these 2524 fish, 1882 individuals were genotyped with the Onil50 SNP-array (see Joshi et al. [5] for details). The raw dataset contained 58,466 single nucleotide polymorphisms (SNPs), which were analysed using the Best Practices workflow of the Axiom Analysis Suite software [20] with default settings (sample Dish QC ≥ 0.82, QC call rate ≥ 97%; SNP call rate cut-off ≥ 97%). Ten samples were below the minimum QC call rate and excluded. Then, SNPs were selected based on informativeness, i.e. on the formation of clusters and resolution. Only those classified as ‘PolyHighResolution’ [20] (formation of three clusters with good resolution) and ‘NoMinorHom’ [20] (formation of two clusters, with one homozygous genotype not present among samples) were selected, resulting in 43,014 SNPs. The mean SNP call rate for these SNPs was 99.5%, ranging from 97 to 100%. Finally, SNPs were filtered for minor allele frequency ≥ 0.05, leaving 39,927 SNPs for analyses (68.3% of the genotyped SNPs). From the marker genotypes, the individual homozygosity ($h_{i}$) was calculated as the proportion of homozygous loci for each individual $i$, and this was added to the models described below as a covariate to account for directional dominance [21, 22].

Of the 1882 genotyped individuals, 1119 individuals from 74 full-sib families had phenotypic records, with an average of 15.1 offspring per full-sib family (ranging from 1 to 44; standard deviation = 11.2), and were used for further analysis. Additional file 1: Tables S1 and S2 show the data structure and descriptive statistics, respectively, while scatterplots and phenotypic correlations among traits are in Additional file 2: Figure S1.

### Statistical analyses

#### Models

ASReml-4 [23] was used to fit mixed linear models, using residual maximum likelihood (REML) to estimate variance components, and breeding values were calculated conditional on the estimated variance components. Eight univariate GBLUP models were tested and compared for the six phenotyped traits. The basic model was an animal model (A), which was gradually expanded to a model with additive (A), dominance (D), maternal (M), and first order epistatic interaction (E) effects (ADME) by adding each effect as a random effect in a heuristic approach. This resulted in the following models:

$${\text{A}}\;{\text{model: }}{\mathbf{y}} = {\mathbf{X}} {\varvec{\upbeta}} + {\mathbf{h}}b + {\mathbf{Z}}_{1}{\mathbf{a}} + {\mathbf{e}},$$

$${\text{AD}}\;{\text{model: }}{\mathbf{y}} = {\mathbf{X}} {\varvec{\upbeta}} + {\mathbf{h}}b + {\mathbf{Z}}_{1} {\mathbf{a}} + {\mathbf{Z}}_{2} {\mathbf{d}} + {\mathbf{e}},$$

$${\text{ADE}}\;{\text{model}}\;{\mathbf{y}} = {\mathbf{X }} {\varvec{\upbeta}} + {\mathbf{h}}b + {\mathbf{Z}}_{1} {\mathbf{a}} + {\mathbf{Z}}_{2} {\mathbf{d}} + {\mathbf{Z}}_{3} {\mathbf{e}}_{\text{aa}} + {\mathbf{e}},$$

$${\text{ADME}}\;{\text{model}}\;{\mathbf{y}} = {\mathbf{X}} {\varvec{\upbeta}} + {\mathbf{h}}b + {\mathbf{Z}}_{1} {\mathbf{a}} + {\mathbf{Z}}_{2} {\mathbf{d}} + {\mathbf{Z}}_{3} {\mathbf{e}}_{\text{aa}} + {\mathbf{Z}}_{4} {\mathbf{m}} + {\mathbf{e}},$$

$${\text{ADM}}\;{\text{model: }}{\mathbf{y}} = {\mathbf{X}} {\varvec{\upbeta}} + {\mathbf{h}}b + {\mathbf{Z}}_{1} {\mathbf{a}} + {\mathbf{Z}}_{2} {\mathbf{d}} + {\mathbf{Z}}_{4} {\mathbf{m}} + {\mathbf{e}},$$

$${\text{AM}}\;{\text{model: }}{\mathbf{y}} = {\mathbf{X}}{\varvec{\upbeta}} + {\mathbf{h}}b + {\mathbf{Z}}_{1} {\mathbf{a}} + {\mathbf{Z}}_{4} {\mathbf{m}} + {\mathbf{e}},$$

$${\text{AME}}\;{\text{model}}\;{\mathbf{y}} = {\mathbf{X}} {\varvec{\upbeta}} + {\mathbf{h}}b + {\mathbf{Z}}_{1} {\mathbf{a}} + {\mathbf{Z}}_{3} {\mathbf{e}}_{\text{aa}} + {\mathbf{Z}}_{4} {\mathbf{m}} + {\mathbf{e}},$$

$${\text{AE}}\;{\text{model: }}{\mathbf{y}} = {\mathbf{X }} {\varvec{\upbeta}} + {\mathbf{h}}b + {\mathbf{Z}}_{1} {\mathbf{a}} + {\mathbf{Z}}_{3} {\mathbf{e}}_{\text{aa}} + {\mathbf{e}},$$

where ${\mathbf{y}}$ is the vector of phenotypic records; $\varvec{\upbeta}$ is the vector of fixed effects, consisting of the effects of reciprocal cross (1 degree of freedom (d.f.)), batch (2 d.f.), and day of harvest (7 d.f.); ${\mathbf{h}}$ is the vector of genomic homozygosity for each individual, with $b$ the regression coefficient, measuring inbreeding depression; ${\mathbf{a}}$ is the vector of random additive genetic effects; ${\mathbf{d}}$ is the vector of random dominance effects; ${\mathbf{e}}_{\text{aa}}$ is the vector of first order additive x additive epistatic effects; ${\mathbf{m}}$ is the vector of random maternal effects; ${\mathbf{e}}$ is the vector of random residual errors; and ${\mathbf{X}}$, ${\mathbf{Z}}_{1}$, ${\mathbf{Z}}_{2}$, ${\mathbf{Z}}_{3}$ and ${\mathbf{Z}}_{4}$, are corresponding design matrices for the fixed and random effects. For FW and FY, the mixed model also included filleter (2 d.f.) as a fixed effect. Vectors ${\mathbf{a}}$, ${\mathbf{d}}$, ${\mathbf{e}}_{\text{aa}}$, and ${\mathbf{e}}$ had an effect for each genotyped individual, where **m** had an effect for each maternal family.

The distributional assumptions for the random effects were multivariate normal, with means zero and

$${\text{Var}}\left[ {\begin{array}{*{20}c} {\mathbf{a}} \\ {\mathbf{d}} \\ {{\mathbf{e}}_{\text{aa}} } \\ {\mathbf{m}} \\ {\mathbf{e}} \\ \end{array} } \right] = \left[ {\begin{array}{*{20}c} {{\mathbf{G}}\upsigma_{\text{A}}^{2} } & 0 & 0 & 0 & 0 \\ 0 & {{\mathbf{D}}\upsigma_{\text{D}}^{2} } & 0 & 0 & 0 \\ 0 & 0 & {{\text{k}}\left( {{\mathbf{G}}\# {\mathbf{G}}} \right)\upsigma_{{{\text{E}}_{\text{aa}} }}^{2} } & 0 & 0 \\ 0 & 0 & 0 & {{\mathbf{I}}\upsigma_{\text{M}}^{2} } & 0 \\ 0 & 0 & 0 & 0 & {{\mathbf{I}}\upsigma_{\text{E}}^{2} } \\ \end{array} } \right],$$

where $\upsigma_{\text{A}}^{2}$, $\upsigma_{\text{D}}^{2}$, $\upsigma_{{{\text{E}}_{\text{aa}} }}^{2}$, $\upsigma_{\text{M}}^{2}$ and $\upsigma_{\text{E}}^{2}$ are the additive genetic, dominance, additive-by-additive epistatic, maternal, and error variance, respectively; ${\mathbf{G}}$ is the genomic relationship matrix with elements ${\text{g}}_{\text{ij}}$; ${\mathbf{D}}$ is the dominance relationship matrix; ${\mathbf{I}}$ is an identity matrix of appropriate size; and ${\text{k}}\left( {{\mathbf{G}}\# {\mathbf{G}}} \right)$ represents the additive-by-additive epistatic relationship matrix, where ${\text{k}}$ is a scaling factor and $\#$ is the Hadamard product, resulting in $\left( {{\mathbf{G}}\# {\mathbf{G}}} \right)_{ij} = {\text{g}}_{\text{ij}}^{2}$ for all elements, following [24–26]. The calculation of elements of ${\mathbf{G}}$, ${\mathbf{D}}$ and ${\text{k}}\left( {{\mathbf{G}}\# {\mathbf{G}}} \right)$ is described in the next section.

The phenotypic variance was calculated as $\upsigma_{\text{P}}^{2} =\upsigma_{\text{A}}^{2} +\upsigma_{\text{D}}^{2} +\upsigma_{{{\text{E}}_{\text{aa}} }}^{2} +\upsigma_{\text{M}}^{2} +\upsigma_{\text{E}}^{2}$, and the estimated variance components were expressed relative to the total phenotypic variance ($\upsigma_{\text{P}}^{2}$): narrow sense heritability $\left( {h^{2} } \right) =\upsigma_{\text{A}}^{2} /\upsigma_{\text{P}}^{2}$, dominance ratio $\left( {d^{2} } \right) =\upsigma_{\text{D}}^{2} /\upsigma_{\text{P}}^{2}$, and maternal ratio $\left( {m^{2} } \right) =\upsigma_{\text{M}}^{2} /\upsigma_{\text{P}}^{2}$. Broad sense heritability ($H^{2}$) was calculated as $(\upsigma_{\text{A}}^{2} +\upsigma_{\text{D}}^{2} +\upsigma_{{{\text{E}}_{\text{aa}} }}^{2} )/\upsigma_{\text{P}}^{2}$. Variance components for terms that were not included in a model were set to 0. For comparison between models the estimated variances were then multiplied by the difference between the mean of the diagonal elements and the mean of all elements of each variance component’s relationship matrix $\left( {\overline{{{\text{diag}}\left( {\mathbf{V}} \right)}} - {\bar{\mathbf{V}}}} \right)$) where ${\mathbf{V}}$ is the corresponding relationship matrix and the bar denotes the mean value [9]).

#### Calculation of relationship matrices

The relationship matrices ${\mathbf{G}}$, ${\mathbf{D}}$ and ${\text{k}}\left( {{\mathbf{G}}\# {\mathbf{G}}} \right)$ were calculated following [12] using two approaches: either assuming HWE (${\mathbf{G}}_{\text{HWE}}$), which has been a standard assumption prior to the work of [12]; or using genomic natural and orthogonal interactions (NOIA), where the assumption of HWE is relaxed. Relaxing the assumption of HWE has consequences for (i) the contrasts between genotypes used to define dominance deviations, and (ii) the scaling factors used for the relationship matrices. However, in both approaches, the contrasts used to define the additive effects are the same (see Additional file 3), so the derivation of ${\mathbf{G}}$ is given first, and in a form that shows continuity with further derivations of ${\mathbf{D}}$ and ${\text{k}}$. The result for ${\mathbf{G}}_{\text{HWE}}$ is identical to Method 1 of VanRaden [7].

Let $x_{ij}$ be the count of an arbitrarily chosen allele for locus $j$ ($j = 1, \ldots ,m$) in individual $i$ ($i = 1, \ldots ,n$), such that $x_{ij}$ = 0, 1 or 2. Additive coefficients $h_{a} \left[ {i,j} \right]$ are assigned for each genotype for each individual, such that $h_{a} \left[ {i,j} \right] = x_{ij} - 2p_{j}$, where $p_{j}$ is the frequency of the chosen allele in the population. These elements $h_{a} \left[ {i,j} \right]$ form an $n \times m$ matrix ${\mathbf{H}}_{a}$, and ${\mathbf{G}}$ for HWE (${\mathbf{G}}_{\text{HWE}}$) and NOIA (${\mathbf{G}}_{\text{NOIA}}$) are calculated as:

$${\mathbf{G}}_{\text{NOIA}} = \left[ {tr\left( {{\mathbf{H}}_{a} {\mathbf{H}}_{a}^{\text{T}} } \right)/{\text{n}}} \right] _{ }^{ - 1} {\mathbf{H}}_{a} {\mathbf{H}}_{a}^{\text{T}} ,$$

$${\mathbf{G}}_{\text{HWE}} = \left[ {2\mathop \sum \limits_{j = 1}^{m} p_{j} \left( {1 - p_{j} } \right)} \right] _{ }^{ - 1} {\mathbf{H}}_{a} {\mathbf{H}}_{a}^{\text{T}} .$$

As noted above and shown in Additional file 3, the contrasts used to form ${\mathbf{H}}_{a}$ for HWE and NOIA are the same but the scaling is different as, in general, without HWE:

$$2\mathop \sum \limits_{j = 1}^{m} p_{j} \left( {1 - p_{j} } \right) \ne tr({\mathbf{H}}_{a} {\mathbf{H}}_{a}^{T} )/n.$$

*Calculating* ${\mathbf{D}}$ *and* ${\text{k}}$ *in the HWE approach.* Both the contrasts and the scaling differ between the HWE and NOIA approaches to calculate ${\mathbf{D}}$. In the HWE approach, dominance coefficients $h_{d} \left[ {i,j} \right]$ are defined for each genotype by orthogonality to the additive coefficients $h_{a} \left[ {i,j} \right]$, assuming the population is in HWE. Therefore, $h_{d} \left[ {i,j} \right] = - 2p_{j}^{2}$, $2p_{j} \left( {1 - p_{j} } \right)$, and $- 2\left( {1 - p_{j} } \right)^{2}$ for $x_{ij}$ = 0, 1, and 2, respectively. The dominance relationship matrix assuming HWE (${\mathbf{D}}_{\text{HWE}}$) is then calculated from the $n \times m$ matrix of coefficients, ${\mathbf{H}}_{d}$, by:

$${\mathbf{D}}_{\text{HWE}} = \left[ {4\mathop \sum \limits_{j = 1}^{m} p_{j}^{2} \left( {1 - p_{j} } \right)^{2} } \right]^{ - 1} {\mathbf{H}}_{d} {\mathbf{H}}_{d}^{T} .$$

In the HWE approach, the relationship matrix for ${\mathbf{e}}_{\text{aa}}$ is the scaled Hadamard product of ${\mathbf{G}}_{\text{HWE}}$ with itself, ${\text{k}}\left( {{\mathbf{G}}_{\text{HWE}} \# {\mathbf{G}}_{\text{HWE}} } \right),$ with ${\text{k}}$ = 1 [12, 27]. If the loci are not in HWE, the relationship matrices ${\mathbf{G}}_{\text{HWE}}$ and ${\mathbf{D}}_{\text{HWE}}$ are not orthogonal to each other and the estimates of the variance components for the additive and dominance effects will depend on whether or not the other set of effects are fitted.

*Calculating* ${\mathbf{D}}$ *and* ${\text{k}}$ *in the NOIA approach.* The NOIA approach removes the assumption of HWE by using dominance values of $h_{d} \left[ {i,j} \right]$ that are calculated such that they are orthogonal to the additive values $h_{a} \left[ {i,j} \right]$, given the observed genotype frequencies. If $p_{j0}$, $p_{j1}$, and $p_{j2}$ denote the genotype frequencies in the population for $x_{ij}$ = 0, 1 or 2, then:

$$h_{d} \left[ {i,j} \right] = \left\{ {\begin{array}{*{20}c} { - 2\left[ {p_{j0} + p_{j2} - \left( {p_{j0} - p_{j2} } \right)^{2} } \right]^{ - 1} p_{j1} p_{j2} } \\ {4\left[ {p_{j0} + p_{j2} - \left( {p_{j0} - p_{j2} } \right)^{2} } \right]^{ - 1} p_{j0} p_{j2} } \\ { - 2\left[ {p_{j0} + p_{j2} - \left( {p_{j0} - p_{j2} } \right)^{2} } \right]^{ - 1} p_{j0} p_{j1} } \\ \end{array} } \right.\;{\text{for}}\;x_{ij} = \left\{ {\begin{array}{*{20}c} 0 \\ 1 \\ 2 \\ \end{array} } \right..$$

The matrix ${\mathbf{D}}_{\text{NOIA}}$ is calculated from the $n \times m$ matrix of coefficients, ${\mathbf{H}}_{d}$, by:

$${\mathbf{D}}_{\text{NOIA}} = [tr\left( {{\mathbf{H}}_{d} {\mathbf{H}}_{d}^{T} } \right)/n]^{ - 1} {\mathbf{H}}_{d} {\mathbf{H}}_{d}^{T} .$$

Note that, in general without HWE, $tr\left( {{\mathbf{H}}_{d} {\mathbf{H}}_{d}^{T} } \right)/n \ne 4\mathop \sum \limits_{j = 1}^{m} p_{j}^{2} \left( {1 - p_{j} } \right)^{2}$.

The relationship matrix for epistatic effects ${\mathbf{e}}_{\text{aa}}$ was calculated from the Hadamard product of ${\mathbf{G}}_{\text{NOIA}}$ with itself (${\mathbf{G}}_{\text{NOIA}} \# {\mathbf{G}}_{\text{NOIA}}$) and scaled by the average of the leading diagonal

$${\text{k}} = \left[ {tr\left( {{\mathbf{G}}_{\text{NOIA}} \# {\mathbf{G}}_{\text{NOIA}} } \right)/n} \right]^{ - 1} {\text{to}}\;{\text{give:}}$$

$$k\left( {{\mathbf{G}}_{\text{NOIA}} \# {\mathbf{G}}_{\text{NOIA}} } \right) = [tr\left( {{\mathbf{G}}_{\text{NOIA}} \# {\mathbf{G}}_{\text{NOIA}} } \right)/n]^{ - 1} {\mathbf{G}}_{\text{NOIA}} \# {\mathbf{G}}_{\text{NOIA}} .$$

Scatterplots for different relationship matrices are presented in Additional file 2: Figures S2 and S3.

The software used to calculate the matrices [12] does not accept missing genotypes. Therefore, missing genotypes (0.4% of all genotypes) were imputed by sampling from {0, 1, 2} using R code [27], with the probabilities for a given SNP given by the population genotype frequencies for that SNP. The effect of this prediction on the relationships between individuals was checked with GCTA [10] by constructing the genomic relationship matrices (GRM) with and without the imputed genotypes. The correlation between the additive and dominance relationships constructed using these two sets of genotypes was greater than 0.9995, as shown on the scatterplots of relationships in Additional file 2: Figure S4. This suggests that use of imputed missing genotypes does not have a significant effect on our results.

### Comparison of models

Likelihood ratio tests (LRT) were used to measure the goodness-of-fit of each model and compared to the LRT of the full model ADME. Critical values for type I error rates of 0.05, 0.01 and 0.001 were corrected for boundary effects when testing the null hypothesis H_0_: $\upsigma^{2}$ = 0 against the alternative hypothesis H_1_: $\upsigma^{2}$ > 0 following [28]. These were obtained from a mixture of $\upchi^{2}$ distributions with different d.f., and calculated using R. The distributions of the likelihood under H_0_ for one, two, and three components were:

$${\raise0.7ex\hbox{$1$} \!\mathord{\left/ {\vphantom {1 2}}\right.\kern-0pt} \!\lower0.7ex\hbox{$2$}}{\text{I}}\left[ 0 \right] + {\raise0.7ex\hbox{$1$} \!\mathord{\left/ {\vphantom {1 2}}\right.\kern-0pt} \!\lower0.7ex\hbox{$2$}}\upchi_{1}^{2} ,\;{\raise0.7ex\hbox{$1$} \!\mathord{\left/ {\vphantom {1 4}}\right.\kern-0pt} \!\lower0.7ex\hbox{$4$}}{\text{I}}\left[ 0 \right] + {\raise0.7ex\hbox{$1$} \!\mathord{\left/ {\vphantom {1 2}}\right.\kern-0pt} \!\lower0.7ex\hbox{$2$}}\upchi_{1}^{2} + {\raise0.7ex\hbox{$1$} \!\mathord{\left/ {\vphantom {1 4}}\right.\kern-0pt} \!\lower0.7ex\hbox{$4$}}\upchi_{2}^{2}$$

and $${\raise0.7ex\hbox{$1$} \!\mathord{\left/ {\vphantom {1 8}}\right.\kern-0pt} \!\lower0.7ex\hbox{$8$}}{\text{I}}\left[ 0 \right] + {\raise0.7ex\hbox{$3$} \!\mathord{\left/ {\vphantom {3 8}}\right.\kern-0pt} \!\lower0.7ex\hbox{$8$}}\upchi_{1}^{2} + {\raise0.7ex\hbox{$3$} \!\mathord{\left/ {\vphantom {3 8}}\right.\kern-0pt} \!\lower0.7ex\hbox{$8$}}\upchi_{2}^{2} + {\raise0.7ex\hbox{$1$} \!\mathord{\left/ {\vphantom {1 8}}\right.\kern-0pt} \!\lower0.7ex\hbox{$8$}}\upchi_{3}^{2}$$, respectively, where $${\text{I}}\left[ 0 \right]$$ corresponds to a point mass of 1 at $${\text{x}} = 0$$.

## Results

### Genetic architecture

The six traits analyzed could be differentiated into three distinct groups based on their likelihood ratio tests for the various models (Table 1): BD and BWH showed evidence of significant maternal environmental effects and of additive-by-additive epistasis. BL and FW showed evidence of significant maternal environmental effects only; whereas BT and FY showed no evidence of either maternal environmental effects or additive-by-additive epistasis. None of the traits showed significant dominance variance. The assumption of HWE in the breeding population did not influence the goodness-of-fit for any model, resulting in identical log likelihood values (results not shown).

**Table 1:** Log likelihood values with significance levels for different models for the six traits

Models	d.f.	BD	BL	BT	BWH	FW	FY
ADME		− 43.48	− 191.28	− 1.78	− 31.51	− 69.90	− 68.55
ADE	1	− 46.55**	− 195.75**	− 2.25	− 35.82**	− 74.74***	− 69.10
ADM	1	− 45.14*	− 192.02	− 2.34	− 33.40*	− 70.40	− 68.65
AME	1	− 43.48	− 191.28	− 1.78	− 31.51	− 69.90	− 68.55
AD	2	− 49.29**	− 197.99***	− 3.04	− 39.29***	− 76.05***	− 69.25
AE	2	− 46.55*	− 195.75**	− 2.25	− 35.82**	− 74.74**	− 69.10
AM	2	− 45.15	− 192.02	− 2.40	− 33.40	− 70.40	− 68.65
A	3	− 49.29**	− 197.99**	− 3.06	− 39.29***	− 76.05**	− 69.25

A model is the basic animal model, which is gradually expanded to an ADME [model with additive (A), dominance (D), maternal (M) and first order additive-by-additive epistatic interactions (E) effects] by adding each effect as random

The six traits are: *BD* body depth, *BL* body length, *BT* body thickness, *BWH* body weight at harvest, *FW* fillet weight, *FY* fillet yield

Significance levels for the likelihood ratio tests are expressed relative to the full model ADME. Critical values for type 1 error rates of 0.05, 0.01 and 0.001 were 2.71, 5.42 and 9.55, respectively, for 1 d.f.; 4.24, 7.29 and 11.77, respectively, for 2 d.f.; and 5.44, 8.75 and 13.48 respectively for 3 d.f. Statistical significance is labelled as ‘*’, ‘**’ and ‘***’ for P < 0.05, P < 0.01 and P < 0.001, respectively

### Inbreeding depression

Detrimental effects of genomic homozygosity were evident for all six traits, although the magnitude differed between traits. BWH and FW were more affected by inbreeding than the other traits, with a 0.01 fractional decrease in the trait per 0.01 increase in the individual homozygosity (shown as “${\text{b}}_{\text{R}}$” in Table 2). The difference between the upper and lower 5%-iles of genomic homozygosity was equal to 0.595 − 0.533 = 0.062 units for the population studied. The impacts of this difference when comparing the upper and lower tails of the population (obtained by multiplying ${\text{b}}_{\text{R}}$ for the trait by the difference between the upper and lower five-percentiles of genomic homozygosity) were: 0.21 cm for BD, 23.21 g for BWH, 0.47 cm for BL, and 9.76 g for FW (shown as “Difference” in Table 2). Traits BT and FY, which showed no evidence of non-additive genetic and maternal environmental effects, were the least affected by genomic homozygosity, with the effects not significantly different from 0 (P > 0.05).

**Table 2:** Estimates of inbreeding depression for six commercial traits in Nile tilapia

	BD	BWH	BL	FW	BT	FY
${\text{b}}$	− 3.27** (1.19)	− 371** (137)	− 7.57* (2.95)	− 156** (56)	− 7.08 (5.05)	− 6.90 (4.93)
Mean	8.89	407.31	22.38	143.83	40.67	32.83
${\text{b}}_{\text{R}}$	0.37	0.91	0.34	1.08	0.17	0.21
Difference	0.21	23.22	0.47	9.76	0.44	0.43
Unit	cm	g	cm	g	mm	%

“${\text{b}}$” is the regression coefficient of the trait on individual homozygosity (trait units per unit fractional homozygosity)

“Mean” is the average value for the studied population (see last line in the table for units)

“${\text{b}}_{\text{R}}$” is the ratio between − b and mean value of the trait

“Difference” is the difference in performance in trait units between the upper and lower 5 percentile for homozygosity in the population, which was 0.062

Standard errors are in parentheses

The six traits are: *BD* body depth, *BWH* body weight at harvest, *BL* body length, *FW* fillet weight, *BT* body thickness, *FY* fillet yield

** Indicates P < 0.01, and * indicates P < 0.05 for significant values

### Decomposition of variance components

**Fig. 1:**
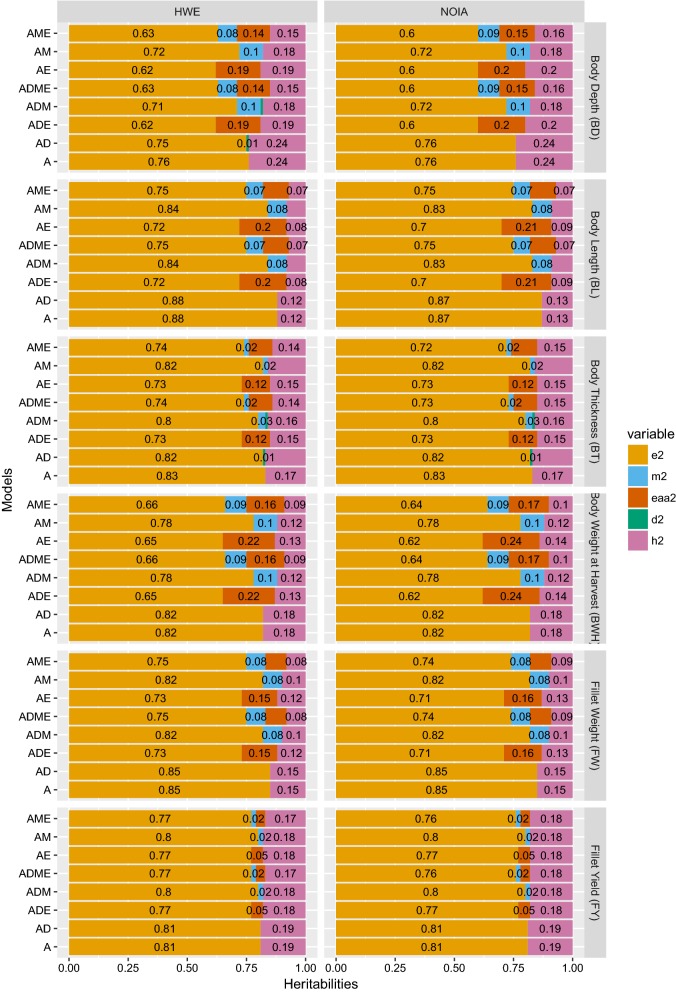
Decomposition of phenotypic variance into different components using approaches based on NOIA and HWE assumption for the six traits. The variance ratios are: $h^{2}$ is additive; $d^{2}$ is dominance; ${\mathbf{e}}_{{{\mathbf{aa}}}}^{2}$ is additive-by-additive epistatic; $m^{2}$ is maternal; and $e^{2}$ is residual

Estimates of variance components with the HWE and NOIA approaches are represented graphically in Fig. 1 for all models and traits. Summary statistics for the models selected based on the LRT are in Table 3.

**Table 3:** Estimates of variance components and their ratios to phenotypic variance for the model of best fit for each trait

Trait	Model	${\text{Additive}}$	${\text{Epistatic}}$	${\text{Maternal}}$	${\text{Residual}}$	${\text{Phenotypic}}$	$h^{2}$	$H^{2}$	$m^{2}$	${\mathbf{e}}_{{{\mathbf{aa}}}}^{2}$
NOIA										
BD	AME	0.086 (0.024)	0.080 (0.049)	0.047 (0.032)	0.328 (0.044)	0.541 (0.039)	0.158 (0.042)	0.307 (0.090)	0.087 (0.055)	0.148 (0.091)
B WH	AME	699 (268)	1183 (680)	635 (418)	4540 (618)	7059 (498)	0.099 (0.037)	0.266 (0.093)	0.090 (0.054)	0.167 (0.096)
BL	AM	0.284 (0.107)		0.257 (0.162)	2.803 (0.136)	3.345 (0.209)	0.085 (0.031)		0.076 (0.045)	
FW	AM	118 (42)		99 (63)	1009 (50)	1227 (79)	0.096 (0.033)		0.080 (0.047)	
BT	A	1.695 (0.441)			8.015 (0.411)	9.710 (0.458)	0.174 (0.041)			
FY	A	1.758 (0.406)			7.461 (0.378)	9.220 (0.435)	0.190 (0.039)			
HWE										
BD	AME	0.097 (0.027)	0.102 (0.063)	0.047 (0.032)	0.326 (0.045)	0.573 (0.042)	0.169 (0.046)	0.348 (0.1)	0.082 (0.053)	0.178 (0.106)
BWH	AME	791 (303)	1504 (864)	635 (418)	4520 (626)	7450 (544)	0.106 (0.04)	0.308 (0.104)	0.085 (0.051)	0.201 (0.111)
BL	AM	0.321 (0.120)		0.257 (0.162)	2.801 (0.136)	3.380 (0.213)	0.095 (0.034)		0.076 (0.044)	
FW	AM	133 (47)		99 (63)	1009 (50)	1241 (81)	0.107 (0.036)		0.079 (0.047)	
BT	A	1.915 (0.498)			8.004 (0.413)	9.92 (0.492)	0.193 (0.044)			
FY	A	1.987 (0.459)			7.450 (0.379)	9.437 (0.467)	0.210 (0.042)			

Standard errors are in parentheses

The estimated ratios are: narrow sense heritability $h^{2}$, broad heritability $H^{2}$, maternal ratio $m^{2}$, and epistatic ratio ${\mathbf{e}}_{{{\mathbf{aa}}}}^{2}$

A model represents the basic animal model, which is gradually expanded to an AME (model with additive (A), maternal (M) and first order additive-by-additive epistatic interactions (E) effects) by adding each effect as random effects

NOIA and HWE are the two approaches used to calculate the different variance components (see “Methods” for details)

The six traits are: *BD* body depth, *BWH* body weight at harvest, *BL* body length, *FW* fillet weight, *BT* body thickness, *FY* fillet yield

The simple A model gave the largest additive genetic variances and the highest narrow sense heritabilities for all traits. Addition of a dominance effect to the models had no effect on the estimates of additive genetic variance, while including additive-by-additive epistatic effects reduced estimates of the additive genetic variance markedly, except for BT and FY, which showed no evidence of epistasis (P > 0.05). Inclusion of maternal environmental effects reduced estimates of the additive genetic variance compared to estimates from the A model, which implies that some of the variance associated with dams was attributed to additive genetic effects in the A model. Including a maternal effect (AME model) also reduced estimates of the additive-by-additive epistatic variance compared to the AE model. As above, these reductions were minimal for BT and FY. The NOIA and HWE approaches resulted in similar estimates of variance components for all models. Thus, in the following, reported estimates are based on the NOIA approach (and so are scaled to the reference population [9]), unless otherwise mentioned.

Model-dependent differences in estimates of additive variance were also reflected in the estimates of narrow sense heritability. For BT and FY, for which the best-fit model was the A model, heritabilities were the least dependent on the model used. For the other traits, the differences in estimates of heritability between models were as large as 50% for some traits. For the best-fit models, the estimates of heritability were low to moderate, ranging from 0.08 ± 0.03 for BL to 0.19 ± 0.04 for FY (Table 4).

**Table 4:** Corrected estimates of heritability, variance ratios, and variances for the model of best fit for each trait and approach

Traits	Models	HWE				NOIA			
		$\upsigma_{\text{A}}^{2}$	$\upsigma_{{{\text{E}}_{{{\mathbf{aa}}}} }}^{2}$	$h^{2}$	${\mathbf{e}}_{{{\mathbf{aa}}}}^{2}$	$\upsigma_{\text{A}}^{2}$	$\upsigma_{{{\text{E}}_{{{\mathbf{aa}}}} }}^{2}$	$h^{2}$	${\mathbf{e}}_{{{\mathbf{aa}}}}^{2}$
BD	AME	0.086 (0.024)	0.080 (0.049)	0.159 (0.043)	0.147 (0.091)	0.086 (0.024)	0.080 (0.049)	0.159 (0.043)	0.147 (0.091)
BWH	AME	698.774 (267.730)	1169.547 (672.154)	0.099 (0.037)	0.167 (0.096)	698.772 (267.729)	1169.539 (672.149)	0.099 (0.037)	0.167 (0.095)
BL	AM	0.285 (0.107)		0.085 (0.031)		0.284 (0.107)		0.085 (0.031)	
FW	AM	117.948 (41.825548)		0.096 (0.0324407)		117.948 (41.825)		0.096 (0.033)	
BT	A	1.694 (0.441)		0.174 (0.041)		1.694 (0.441)		0.174 (0.041)	
FY	A	1.757 (0.406)		0.191 (0.039)		1.758 (0.406)		0.191 (0.039)	

The variances and ratios using the HWE approach were corrected by (mean (leading diagonal) − mean) of the the corresponding relationship matrices as per [9]

The mean of diagonal and off-diagonal elements in relationship matrices using the NOIA approach resulted ~ 1 and 0, respectively. Hence, scaling was not required for the NOIA approach

Standard errors are in parentheses

A model represents the basic animal model, which is gradually expanded to an AME (model with additive (A), maternal (M) and first order additive-by-additive epistatic interactions (E) effects) by adding each effect as random effects

NOIA and HWE are the two approaches used to calculate the different variance components (see “Methods” for details)

The six traits are: *BD* body depth, *BWH* body weight at harvest, *BL* body length, *FW* fillet weight, *BT* body thickness, *FY* fillet yield

**Fig. 2:**
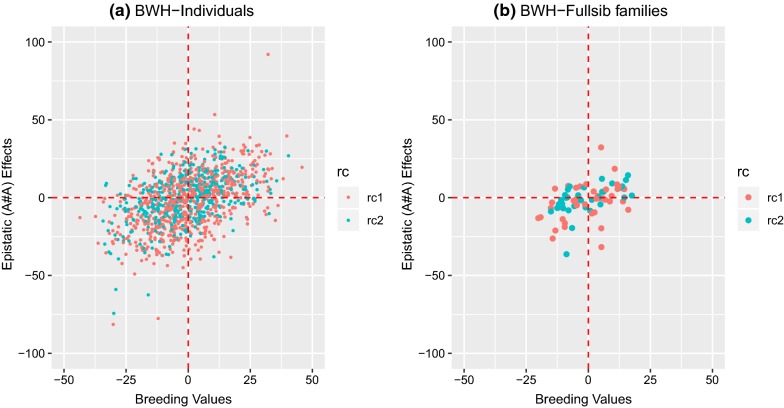
Scatterplot of estimated breeding values (EBV) and epistatic (additive-by-additive) values (EEV) for BWH using the NOIA approach; **a** scatterplot for all individuals; **b** scatterplot for means of full-sib families. Color of the dots in the scatterplot represents the reciprocal cross (rc): A × B (rc1) and B × A (rc2)

For BD and BWH, the traits for which the best-fit model included additive-by-additive epistasis, the additive-by-additive epistasis ratio (${\text{e}}_{\text{aa}}^{2}$) was equal to 0.15 ± 0.09 and 0.17 ± 0.10 (Table 4), respectively, and the variance represented 48 and 63% of the total genetic variance, respectively, but with large standard errors. Various other papers with genomic epistatic models also report large epistatic components [12, 29, 30] with corresponding large standard errors. For predicted additive-by-additive epistatic effects, large differences were observed between individuals (Fig. 2a) and between full-sib families (Fig. 2b). The models were further extended by fitting additive × dominance (${\mathbf{e}}_{\text{ad}}$) and dominance × dominance (${\mathbf{e}}_{\text{dd}}$) epistatic effects, separately and in combination with ${\mathbf{e}}_{\text{aa}}$. The estimates of variance associated with ${\mathbf{e}}_{\text{ad}}$ and ${\mathbf{e}}_{\text{dd}}$ were zero, and estimates of all other components were identical to those of the models described above (results not shown).

For the four traits for which the best-fit model included a maternal environmental effect, the maternal ratio ranged from 0.08 ± 0.04 to 0.09 ± 0.06. As expected, this variance ratio was not affected by the approaches (NOIA, HWE).

## Discussion

### Interpretation of the variance within full-sib families

A major finding of this study is that the use of genomic relationship matrixes found non-additive genetic variance to be almost entirely represented by additive-by-additive epistasis. In pedigree-based analyses, the primary source of non-additive variance is commonly assumed to be dominance [1, 31, 32], but this can be very misleading, as is the case here, where estimates of dominance variance were negligible. In our study, information for estimating non-additive variance comes from the variance within full-sib families [see Additional file 4], and with presence of both dominance and epistasis, the additional variance expected in full-sib families over and above the additive variance from the sire and dam is $${\raise0.7ex\hbox{$1$} \!\mathord{\left/ {\vphantom {1 4}}\right.\kern-0pt} \!\lower0.7ex\hbox{$4$}}\upsigma_{{\mathbf{D}}}^{2}+ {\raise0.7ex\hbox{$1$} \!\mathord{\left/ {\vphantom {1 8}}\right.\kern-0pt} \!\lower0.7ex\hbox{$8$}}\upsigma_{{{\mathbf{E}}_{\text{aa}} }}^{2} + {\raise0.7ex\hbox{$1$} \!\mathord{\left/ {\vphantom {1 8}}\right.\kern-0pt} \!\lower0.7ex\hbox{$8$}}\upsigma_{{{\mathbf{E}}_{\text{ad}} }}^{2} + {\raise0.7ex\hbox{$1$} \!\mathord{\left/ {\vphantom {1 8}}\right.\kern-0pt} \!\lower0.7ex\hbox{$8$}}\upsigma_{{{\mathbf{E}}_{\text{dd}} }}^{2}$$, where $$\upsigma_{{\mathbf{D}}}^{2}$$, $$\upsigma_{{{\mathbf{E}}_{\text{aa}} }}^{2}$$,$$\upsigma_{{{\mathbf{E}}_{\text{ad}} }}^{2}$$ and $$\upsigma_{{{\mathbf{E}}_{\text{dd}} }}^{2}$$ are dominance, additive-by-additive, additive-by-dominance, and dominance-by-dominance epistatic variances [33]. Under a polygenic model with both additive and dominance effects and an increasing number of loci, either the dominance variance tends towards zero or the inbreeding depression tends towards infinity [33, 34]. Thus, dominance may be present, but the genomic approach shows that this component behaves infinitesimally, with $$\upsigma_{{\mathbf{D}}}^{2}$$, $$\upsigma_{{{\mathbf{E}}_{\text{ad}} }}^{2}$$ and $$\upsigma_{{{\mathbf{E}}_{\text{dd}} }}^{2}$$ being undetectable in the analyses.

### Comparison with the pedigree approach

The findings from this study add a new dimension to our previous paper [1]. The availability of genomic data in populations inevitably leads to comparisons between genomic- and pedigree-based heritabilities, but these are not straightforward. Some studies argue that pedigree-based methods overestimate heritabilities [35–37], while some suggest the reverse [38–41], and another that they are similar [42]. However, few studies recognize that the variance estimates obtained from models that use different relationship matrices, even for the basic additive models, do not refer to the same populations, making simple comparison of estimates meaningless. For pedigree-based analyses, variance estimates refer to the base population of the pedigree (a subset of the individuals in the numerator relationship matrix, **A**), while for genomic-based analyses using Van Raden matrices [7] the estimates refer to the population included in the ${\mathbf{G}}$ matrix, assuming that all markers are in HWE. Only after correcting for the different reference populations [9, 12] can objective comparisons be made. Here the estimated genetic variance within the entire study population was used for comparing results from different relationship matrices, and was obtained by multiplying the variance estimates by $\overline{{{\text{diag}}\left( {\mathbf{V}} \right)}} - {\mathbf{V}}$, as described in “Methods” and [9]. For NOIA estimates this multiplication factor is 1 because of the method used to construct ${\mathbf{V}}$.

When models include non-additive genetic components, there are additional reasons why estimates of variance components can differ between models using different relationship matrices (e.g. pedigree and genomic models). In the tilapia population studied here, the estimate of additive variance is qualitatively different when the source of non-additive variation is assumed to be dominance instead of additive epistasis (see Additional file 4). Therefore, results from this study are expected to be different from those of [1]. In addition, only a subset of the data from [1] was used, although Additional file 2: Figure S5 shows that the data used here was close to expectations from random sampling. To aid comparability, model A was fitted using the pedigree-based relationship matrix for the current subset (see Additional file 1 Table S4). The outcome shows that the pedigree-based and genomic analyses had a qualitatively similar pattern of contributing sources of variance, insofar as additive, maternal, and non-additive variances, for all six traits. Evidence of non-additive genetic effects was found for the same traits (BD, BWH), irrespective of the type of relationships used. However, as mentioned above, the genomic approach identified the source of non-additivity as additive-by-additive epistasis rather than dominance.

Genomic models were more robust to misspecification in partitioning the variance among the components of the genetic and environmental models, which is another potential cause of differences between genomic and pedigree-based models. The greater robustness of the genomic model was clearly observed when the A model was fitted to traits for which the genetic architecture was found to be more complex (results are in Additional file 1: Table S4). In the A model, maternal effects were absorbed into the estimate of the additive variance when using pedigree data, in contrast to the genomic model, for which the genotypes of the dam and its offspring contribute information to the estimate of additive genetic variance, such that maternal effects are no longer (wrongly) absorbed into the additive variance. Hence, pedigree-based estimates of heritability based on the A model were higher for traits with maternal effects, by as much as 0.18.

### Impact of HWE and NOIA approaches on variance components

GBLUP uses a genomic relationship matrix, ${\mathbf{G}}$, and assumptions in the construction of ${\mathbf{G}}$ can have a direct effect on estimates of variance components. For example, the ${\mathbf{G}}$ proposed by VanRaden [7], assume HWE when scaling the relationship matrices, while this assumption is avoided with ${\mathbf{G}}$ based on the NOIA approach. In this study, the use of the HWE and NOIA approaches for constructing genomic relationship matrices had no impact on the qualitative outcomes related to the genetic architecture of the trait, but did have a quantitative effect on estimates of variance components, e.g., the estimate of the additive-by-additive epistatic ratio (${\mathbf{e}}_{\text{aa}}^{2}$) was inflated by about 20 and 18%, and the estimate of heritability by 6 and 10% for BD and BWH, respectively (Table 3). Such quantitative differences in estimates between the HWE and NOIA approaches have also been observed in other studies [12]. Because of the absence of dominance variance for the traits studied in this population, differences between the NOIA and HWE approaches collapsed into differences in scaling of the relationship matrices since the contrasts used to construct the matrices were identical. As a result, the transformation of estimates of variance components from HWE to a similar scale as the NOIA approach by multiplying estimates by $\overline{{{\text{diag}}\left( {\mathbf{V}} \right)}} - {\bar{\mathbf{V}}}$ for the corresponding relationship matrices yielded identical estimates of variance components and ratios for the HWE and NOIA approaches.

The NOIA and HWE approaches are statistical models in that they partition the variance observed in a population and use the resulting estimates of variance components to estimate breeding values and dominance deviations [12]. As such, the latter estimates depend on allele frequencies in the population and on its mating structure, which will influence genotype frequencies. A distinction must be made between the magnitudes of the variance components contributing to the total genetic variance and the size of the effects for each genotype, which are the basis of biological models [43, 44]. For example, genotypes at a locus may show complete dominance but have negligible dominance deviations, because the superior homozygote is very rare in the population. Although the NOIA approach removes the limitations of the HWE assumption, major barriers to building biological models remain. First, the NOIA approach does not remove the impact of linkage disequilibrium on the estimates of effects and, more seriously, the biological models are meaningful only if they are constructed with the causal variants but not when using anonymous markers.

### Inbreeding depression

Absence of dominance variance does not imply absence of inbreeding depression when the genetic architecture of a trait approaches the infinitesimal model, and we found evidence for inbreeding depression in precisely the four traits for which the A model was rejected. To the best of our knowledge, these estimates of inbreeding depression are the first reported for commercial traits in Nile tilapia. Most of the previous quantifications of inbreeding depression were based on pedigree information in other aquaculture species, e.g. [45–47], and a few were based on genomic data, e.g. [48]. In this study, estimation was not possible without the application of genomics because of the near identical pedigree-based inbreeding coefficients among individuals in the population. Most of the traits clearly showed inbreeding depression and, if inbreeding depression was ignored in the model, estimates of variance components and estimates of breeding values can become biased (see Additional file 5). Furthermore, inbreeding depression is important in commercial production: for example, FW decreased by 1% per 1% increase in homozygosity and the difference between the upper and lower 5 percentiles for homozygosity in the studied population was 6%, corresponding to a 6% difference in FW. Homozygosity can be minimized by controlling inbreeding or by crossing unrelated lines. The latter will cause a large reduction in inbreeding depression if the regression on homozygosity also holds across lines.

In a polygenic model, allelic additive effects ($a^{\prime}$) are of the order of $1/\sqrt m$ (i.e. ${\text{O}}(1/\sqrt {m)}$), as the number of loci ($m$) becomes large, such that the additive variance remains finite. For inbreeding depression to remain finite, directional dominance deviations ($d^{\prime}$) must be ${\text{O}}\left( {1/m} \right)$. Therefore, a consequence of a polygenic dominance model is that $d^{\prime}/a^{\prime}$ must reduce by $1/\sqrt m$ as $n$ increases. This is consistent with biological pathway models, such as in [49], since for loci that have an increasingly small effect, metabolic responses will be more adequately described by a linear function based on the gradient of the response, such that the importance of partial dominance will decrease.

### Use of the additive-by-additive epistatic effects

In the long term, additive-by-additive epistatic variance is expected to be exploited indirectly because it is converted to additive genetic variance due to random drift and selection. As a result, additive-by-additive epistatic variance affects medium and long-term selection response indirectly [50]. This argues for the use of a simple breeding scheme that uses only additive genetic effects. However, re-structuring towards a cross-breeding scheme, e.g. reciprocal recurrent selection, may be desirable because of the inbreeding depression and the infinitesimal dominance detected, or the maternal effects if part of this variation was found to be heritable.

Nevertheless, for some traits, substantial additive-by-additive epistasis was observed although epistatic variance is expected to be much smaller than the additive genetic variance in elite commercial populations [33, 50]. This raises two questions. First, should epistatic effects be included when estimating genetic parameters? Doing so likely does not benefit selection decisions, partly because the additive genetic variance already contains some of the variance arising from epistatic effects [29, 33, 51]. Second, can the large epistatic variance ratio that was observed for some traits in the Nile tilapia population, which predicts large differences in epistatic values between individuals (Fig. 2), be capitalized on, in some way, in the Nile tilapia breeding program? While our estimates of epistasis rely upon anonymous loci, a more direct exploitation of epistasis would require identification of the causal variants showing large epistatic interactions [52, 53] for each trait. The latter will require substantial resources, probably an order of magnitude greater than resources required for identifying causal variants with additive effects. Hence, this approach seems complex and costly to realize.

## Conclusions

Our findings show that the non-additive genetic variance in the Nile tilapia population was almost entirely additive-by-additive epistatic variance, when using genomic relationship matrices, while these non-additive effects were found to be associated with dominance when using pedigree-based relationship matrixes. Presence of inbreeding depression and lack of dominance variance were consistent with an infinitesimal dominance model for the studied traits. Finally, creating maternal lines in Tilapia breeding schemes may be desirable if the observed maternal variance is heritable.

## Supplementary information

**Additional file 1: Table S1.** Number of animals genotyped in different full-sib families. **Table S2.** Descriptive statistics for the six traits. **Table S3.** Mean values of the genomic relationship matrices constructed with NOIA and HWE approaches. **Table S4.** Transformation of the variances on a similar scale based on the relationship matrices.

**Additional file 2: Figure S1.** Scatterplots, histograms and the correlations of the 6 traits studied. **Figure S2.** Scatterplots for different additive and dominance relationships. **Figure S3.** Scatterplots for different additive, dominance and epistasis relationships using NOIA and HWE approaches. **Figure S4.** Scatterplot and correlation of the additive and dominance relationships using imputed and without imputed genotypes. **Figure S5.** Density plots, scatterplot and LOESS regression between the selected and non-selected individuals.

**Additional file 3.** Equivalence of contrasts used to construct **H**_a_ for HWE and NOIA genomic relationship matrices. The file presents the proof that the difference between ${\mathbf{G}}_{\text{HWE}}$ and ${\mathbf{G}}_{\text{NOIA}}$ lies only in their scaling and not in the contrasts used to calculate the elements of ${\mathbf{H}}_{\text{a}}$.

**Additional file 4.** Assumptions on the nature of non-additive genetic variance and the impact on estimates of additive genetic variance. The file presents the numerical proof that the estimate of additive genetic variance is reduced when the non-additive variation is assumed to be additive-by-additive epistasis rather than dominance, and this reduction is of the order of 3/8 epistatic variance.

**Additional file 5.** Impact of inbreeding depression on models. The file contains the tables with the models without individual homozygosity as the covariate to account for the impact of the inbreeding depression in the models.

## Data Availability

The genotype data used in the study are from commercial family material. This information may be made available to non-competitive interests under conditions specified in a Data Transfer Agreement. Requests to access these datasets should be directed to Alejandro Tola Alvarez: alex@genomar.com.
