# Supplementary material for: Genomic dissection of maternal, additive and non-additive genetic effects for growth and carcass traits in Nile tilapia
Source: Genet Sel Evol. 2020 Jan 15;52:1. doi: 10.1186/s12711-019-0522-2 (PMC6964056; doi:10.1186/s12711-019-0522-2)
Supplement: Supplementary file 1 — Additional file 1: Table S1. Number of animals genotyped in different full-sib families. Table S2. Descriptive statistics for the six traits. Table S3. Mean values of the genomic relationship matrices constructed with NOIA and HWE approaches. Table S4. Transformation of the variances on a similar scale based on the relationship matrices. [file 12711_2019_522_MOESM1_ESM.pdf]

Additional file 1: Supplementary Tables

Genomic dissection of maternal, additive and non-additive genetic effects for growth and carcass traits in Nile tilapia

R Joshi, THE Meuwissen, JA Woolliams and HM Gjøen

Table S1: Number of animals genotyped in different full-sib families.

		1	2	3	4	5	6	7	8	9	10	11	12	13	14	15	16	17	18
**1**	**P**				5	9		30		5	5	26	26		34		15	35	
	**G**																		
**2**	**P**	30	20	18			11		33					9		3			16
	**G**	30	20	18			11							8					
**3**	**P**	29	18	25			6		36					8		13			36
	**G**																		
**4**	**P**	13	7	19			6		23					11		6			14
	**G**																		
**5**	**P**							6		8	7	20	12		25		10	27	
	**G**							6		8	7	20			21		10	27	
**6**	**P**				3	6		46		8	4	39	44		59		17	54	
	**G**							41		8	4	39			38		17	44	
**7**	**P**	32	9	17			8		32					7		2			30
	**G**	32	9	17			8							7					
**8**	**P**				5	3		24		9	3	13	13		19		8	16	
	**G**							25		8	3	13			10		8	16	
**9**	**P**	29	16	26			9		37					11		3			27
	**G**	29	15	26			9							11					
**10**	**P**	35	27	16			8		38					17		6			30
	**G**																		
**11**	**P**				1	2		13		2	1	8	13		22		8	21	
	**G**																		
**12**	**P**				4	2		36		4	3	10	30		47		10	52	
	**G**							36		4	3	10			34		8	43	
**13**	**P**	19	3	14			3		19					7		1			10
	**G**	19	3	14			3							7					
**14**	**P**				2	6		14				16	13		15		12	45	
	**G**							14				16			9		12	43	
**15**	**P**	16	15	22			6		28					12		4			29
	**G**	16	15	20			6		1					12					
**16**	**P**	22	13	17			4		26					15		3			17
	**G**	22	13	17			5		1					16					1
**17**	**P**					4		17		4	6	17	17		34		7	4	
	**G**																		
**18**	**P**							14		6	3	12	22		15		8	18	
	**G**							14		6	3	12	1		13		7	17	

The rows with P and G denote the total number of phenotyped (coded black) and genotyped animals (coded blue) respectively from each full-sib family. Parents are coded from 1 to 18 in the header rows and columns. Empty cells mean no phenotypes or genotypes were available for that full-sib family.

Table S2: Descriptive statistics for the six traits.

	N	Unit	Min	Max	Median	Mean (SE)	SD	CV%
**BWH**	1119	g	115.60	802.80	390.20	407.31 (3.84)	128.44	31.53
**BL**	1119	cm	14.10	28.00	22.40	22.38 (0.07)	2.25	10.05
**BD**	1119	cm	5.00	12.00	8.80	8.89 (0.03)	1.03	11.58
**BT**	1119	mm	12.90	59.70	40.50	40.70 (0.14)	4.55	11.17
**FW**	1119	g	20.10	342.60	136.60	143.83 (1.56)	52.32	36.38
**FY**	1119	%	15.24	50.53	33.15	32.83 (0.09)	3.13	9.54

N is the number of observations having both phenotypes and genotypes, SD is the standard deviation, SE is the standard error and CV is the coefficient of variation expressed as percentage.

Table S3: Mean values of the genomic relationship matrices constructed with NOIA and HWE approaches.

	HWE			NOIA		
	Overall	Diagonal	Off-diagonal	Overall	Diagonal	Off-diagonal
**G**	0.000790	0.8847333	-0.000791354	0.000893	1	-0.000894454
**D**	0.038489	0.9250984	0.036903020	0.000893	1	-0.000894454
**k(G#G)**	0.009216	0.7868253	0.007825131	0.011713	1	0.009945195
**k(G#D)**	0.002971	0.8211928	0.001507525	0.003311	1	0.001528032
**k(D#D)**	0.005130	0.8581108	0.003604039	0.004223	1	0.002441192

All relationship matrices are calculated from the 1119 individuals in the study population.

Table S4: Transformation of the variances on a similar scale based on the relationship matrices.

Traits	Assumption	σ^2_A	SE	σ^2_A*	SE	h^2	SE	h^{2*}	SE
BWH	NOIA	1242	300	1241	300	0.18	0.04	0.19	0.04
	HWE	1404	339	1241	300	0.20	0.04	0.19	0.04
	Ped	3489	1391	2527	1007	0.44	0.14	0.36	0.10
BD	NOIA	0.13	0.03	0.13	0.03	0.24	0.04	0.24	0.04
	HWE	0.14	0.03	0.13	0.03	0.27	0.05	0.24	0.04
	Ped	0.31	0.12	0.23	0.09	0.50	0.15	0.42	0.11
BL	NOIA	0.40	0.12	0.40	0.12	0.13	0.04	0.13	0.04
	HWE	0.46	0.14	0.40	0.12	0.14	0.04	0.13	0.03
	Ped	1.20	0.50	0.87	0.36	0.33	0.12	0.26	0.08
FW	NOIA	177	47	177	47	0.15	0.04	0.16	0.04
	HWE	200	53	177	47	0.17	0.04	0.16	0.04
	Ped	536	215	388	156	0.39	0.13	0.32	0.09
BT	NOIA	1.70	0.44	1.69	0.44	0.17	0.04	0.18	0.04
	HWE	1.92	0.50	1.69	0.44	0.19	0.04	0.18	0.04
	Ped	1.83	0.92	1.32	0.67	0.18	0.08	0.14	0.06
FY	NOIA	1.76	0.41	1.76	0.41	0.19	0.04	0.19	0.04
	HWE	1.99	0.46	1.76	0.41	0.21	0.04	0.19	0.04
	Ped	2.65	1.17	1.92	0.85	0.26	0.10	0.21	0.07

The additive genetic variance (σ^2_A) and the heritability (h^2) were obtained from the A model, but with no individual homozygosity fitted. The transformed variances and ratio are marked by *, and were obtained from scaling estimates by ($\overline{\mathrm{diag}(\mathbf{V})} - \bar{\mathbf{V}}$) of the corresponding relationship matrix **V** (see Table S3) as per [1]. In "Ped", the genomic relationship matrix was replaced by the numerator relationship matrix obtained from 3 generations of pedigree (**A**). $\bar{\mathbf{A}}$ and $\overline{\mathrm{diag}(\mathbf{A})}$ for the 1119 individuals in the study population were 0.2757587 and 1 respectively.

Literature cited:
1. Legarra A. Comparing estimates of genetic variance across different relationship models. Theor Popul Biol. Elsevier; 2016;107:26–30.
