# Supplementary material for: Genomic dissection of maternal, additive and non-additive genetic effects for growth and carcass traits in Nile tilapia
Source: Genet Sel Evol. 2020 Jan 15;52:1. doi: 10.1186/s12711-019-0522-2 (PMC6964056; doi:10.1186/s12711-019-0522-2)
Supplement: Supplementary file 2 — Additional file 2: Figure S1. Scatterplots, histograms and the correlations of the 6 traits studied. Figure S2. Scatterplots for different additive and dominance relationships. Figure S3. Scatterplots for different additive, dominance and epistasis relationships using NOIA and HWE approaches. Figure S4. Scatterplot and correlation of the additive and dominance relationships using imputed and without imputed genotypes. Figure S5. Density plots, scatterplot and LOESS regression between the selected and non-selected individuals. [file 12711_2019_522_MOESM2_ESM.pdf]

Additional file 2: Supplementary Figures

Genomic dissection of maternal, additive and non-additive genetic effects for growth and carcass traits in Nile tilapia

R Joshi, THE Meuwissen, JA Woolliams and HM Gjøen

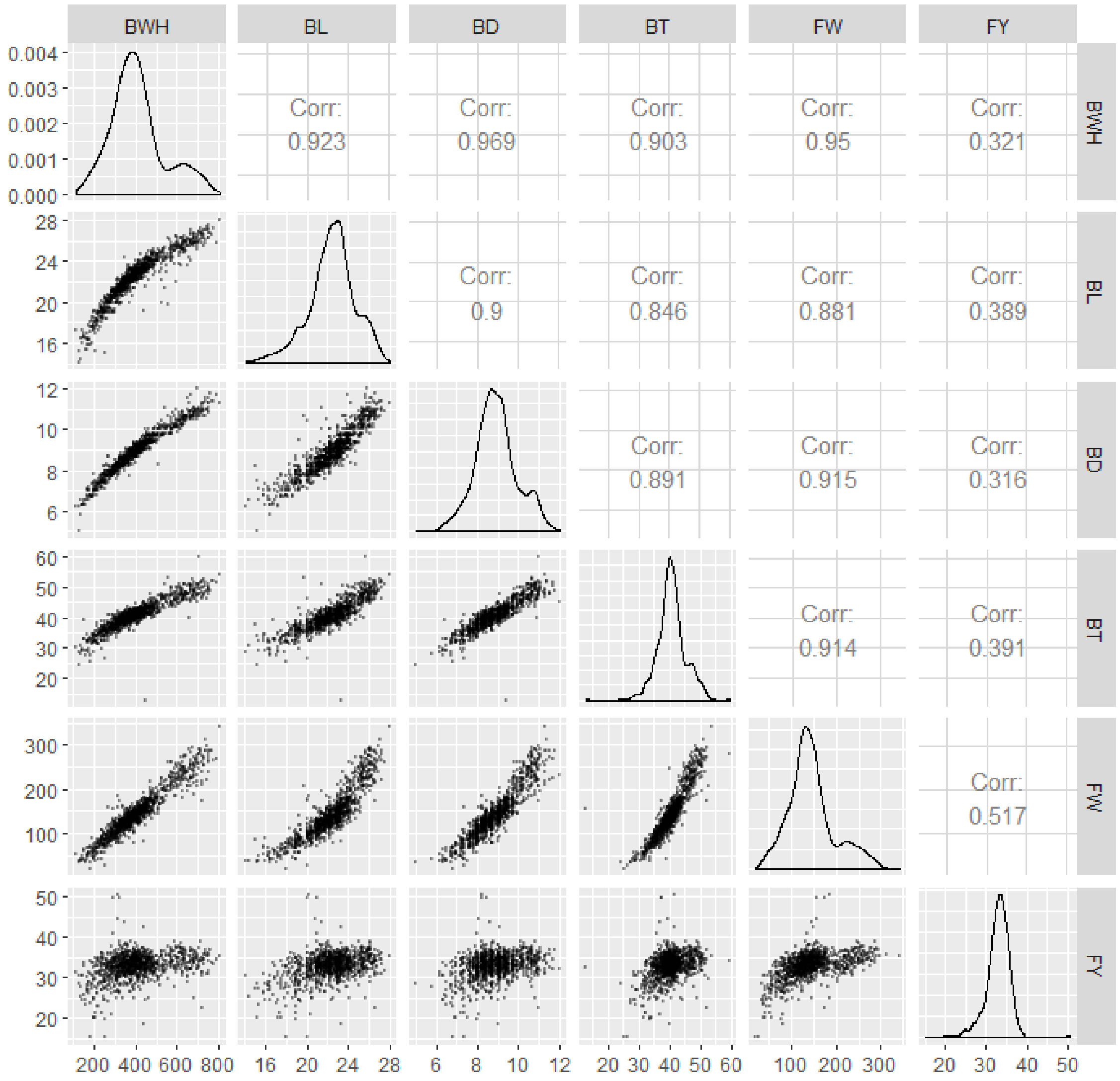

Figure S1: Scatterplots, histograms and the phenotypic correlations for the 6 traits studied.

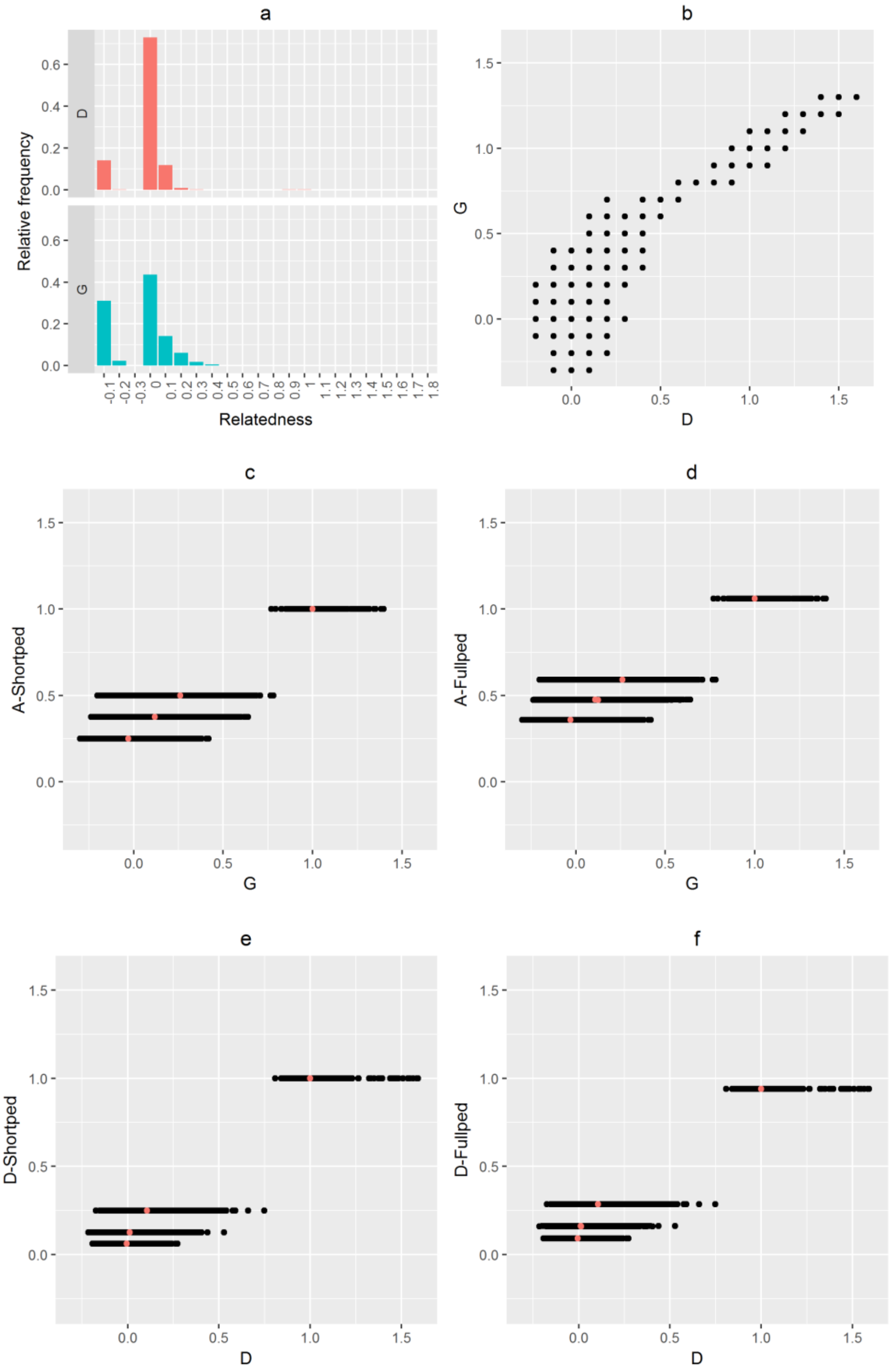

Figure S2: Scatterplots for different additive and dominance relationships. **a)** Relative frequency plots for genomic relationships (**G**$_{\mathbf{NOIA}}$) and dominance relationships using genomic information (**D**$_{\mathbf{NOIA}}$). **b)** Scatterplot of **G**$_{\mathbf{NOIA}}$ with **D**$_{\mathbf{NOIA}}$ (correlation = 0.47). **c)** Scatterplot of **G**$_{\mathbf{NOIA}}$ with the pedigree relationships using 3 generations of pedigree (A-Shortped) (correlation = 0.72). **d)** Scatterplot of **G**$_{\mathbf{NOIA}}$ with the pedigree relationships using 20 generations of pedigree (A-Fullped) (correlation = 0.72). **e)** Scatterplot of **D**$_{\mathbf{NOIA}}$ with the dominance relationships using 3 generations of pedigree (D-Shortped) (correlation =0.65). **f)** Scatterplot of **D**$_{\mathbf{NOIA}}$ with the dominance relationships using 20 generations of pedigree (D-Fullped) (correlation = 0.62). Mean values of **G**$_{\mathbf{NOIA}}$ and **D**$_{\mathbf{NOIA}}$ are shown as orange dots in the respective plots.

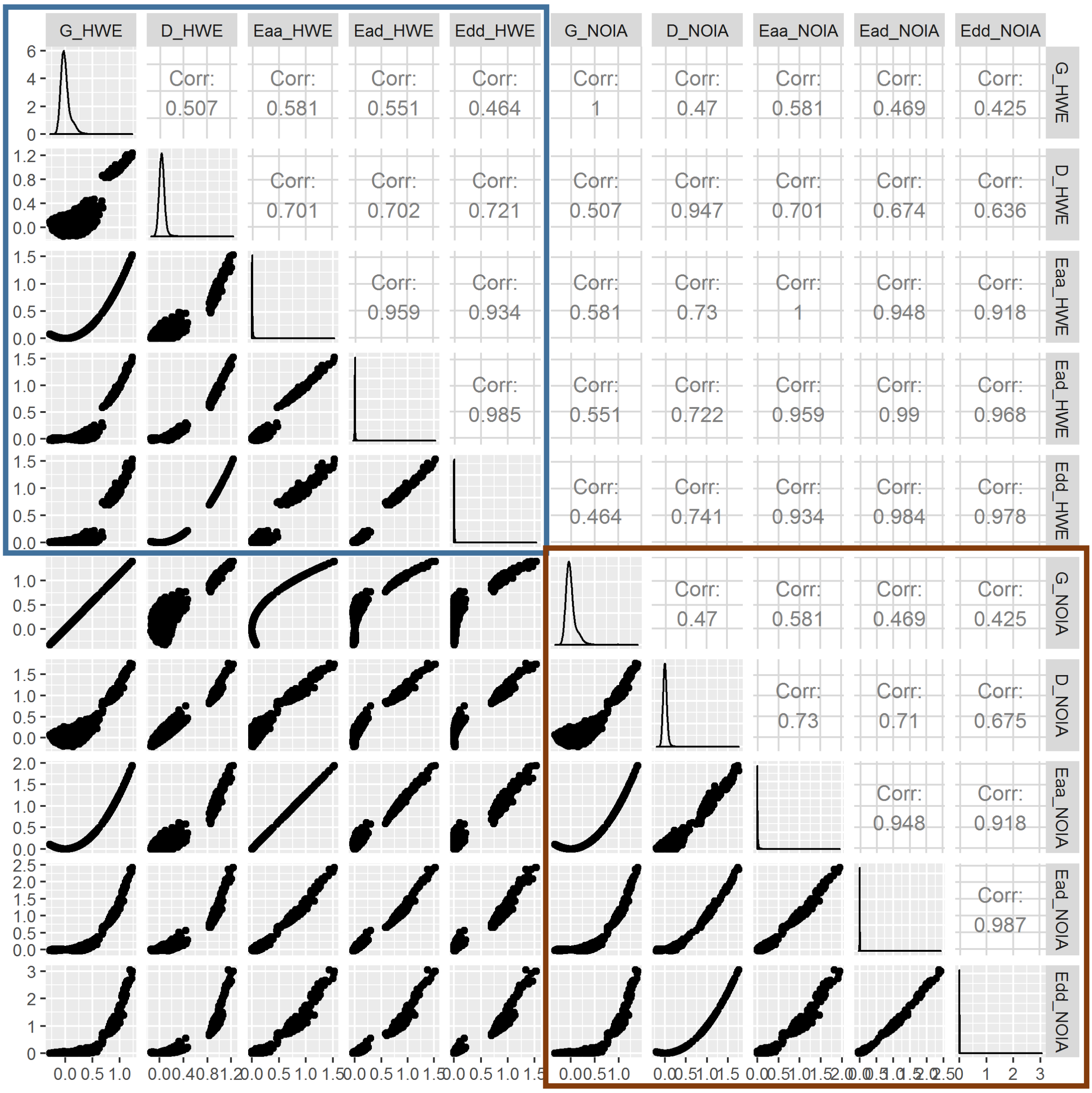

Figure S3: Scatterplots for different additive, dominance and epistasis relationships using NOIA (inside brown box) and HWE approaches (inside blue box).

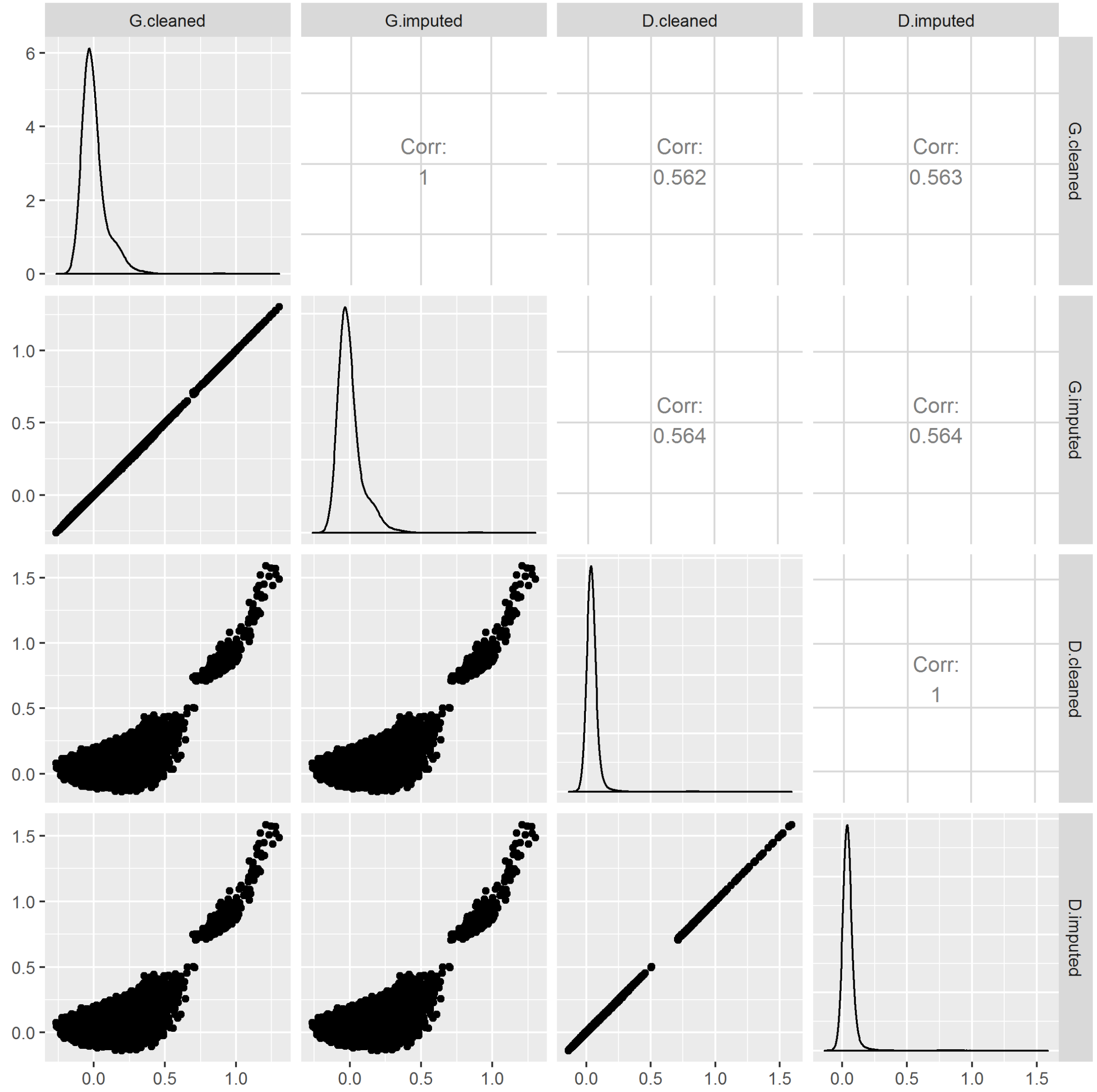

Figure S4: Scatterplot and correlation of the additive and dominance relationships using imputed ('G.imputed' and 'D.imputed' respectively) and without imputed genotypes, i.e. with some missing genotypes ('G.cleaned' and 'D.cleaned' respectively).

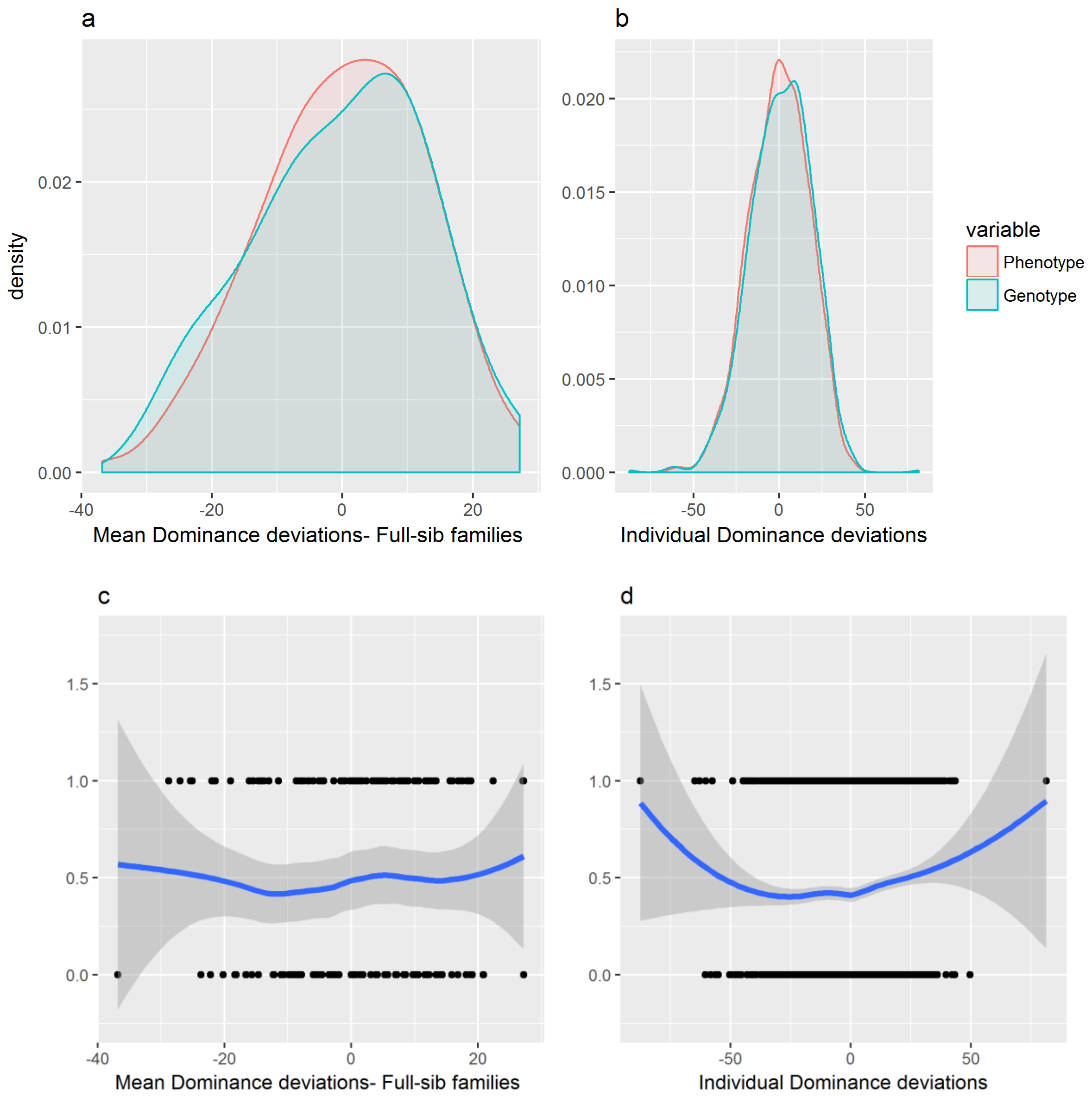

Figure S5: Density plots, scatterplot and LOESS regression between the selected and non-selected individuals. **a)** and **b)** are density plots showing the dominance deviations for BWH between the 2524 animals having phenotypes, and the subset of 1119 genotyped individuals included in this study. **c)** and **d)** are scatterplots showing LOESS regressions. In **c)** the 74 full-sib families represented in this study are coded as 1, and the remaining 81 families are coded as 0 and are plotted against the mean family dominance deviation. In **d)** the 1119 individuals included in this study were coded as 1 and the remainder were coded as 0 and are plotted against individual dominance deviations. The dominance deviations were obtained from the ADM model given in [1].

Literature cited

1. Joshi R, Woolliams JA, Meuwissen THE, Gjøen HM. Maternal, dominance and additive genetic effects in Nile tilapia; influence on growth, fillet yield and body size traits. Heredity (Edinb). 2018;120:452-62.
