# Supplementary material for: Genomic dissection of maternal, additive and non-additive genetic effects for growth and carcass traits in Nile tilapia
Source: Genet Sel Evol. 2020 Jan 15;52:1. doi: 10.1186/s12711-019-0522-2 (PMC6964056; doi:10.1186/s12711-019-0522-2)
Supplement: Supplementary file 3 — Additional file 3. Equivalence of contrasts used to construct Ha for HWE and NOIA genomic relationship matrices. The file presents the proof that the difference between ${\mathbf{G}}_{\text{HWE}}$GHWE and ${\mathbf{G}}_{\text{NOIA}}$GNOIA lies only in their scaling and not in the contrasts used to calculate the elements of ${\mathbf{H}}_{\text{a}}$Ha. [file 12711_2019_522_MOESM3_ESM.pdf]

Additional file 3: Equivalence of contrasts used to construct $\mathbf{H}_a$ for HWE and NOIA genomic relationship matrices

Genomic dissection of maternal, additive and non-additive genetic effects for growth and carcass traits in Nile tilapia

R Joshi, THE Meuwissen, JA Woolliams and HM Gjøen

Let x_{ij} be the count of an arbitrarily chosen alternative allele for locus *j* (*j* = 1 … m) in individual *i* (i = 1 … n) so that x_{ij} = 0, 1 or 2. Following the notation of [1], the genomic relationship matrices for additive effects are constructed from contrasts h_a to give coefficients $h_a[i, j]$ for each locus for each individual and the $h_a[i, j]$ form an *n* x *m* matrix **Ha**. **G** is then calculated as

$$\mathbf{G}_{\mathrm{NOIA}} = [tr(\mathbf{H}_{\mathrm{a}}\mathbf{H}_{\mathrm{a}}^{\mathrm{T}})/\mathrm{n}]^{-1}\mathbf{H}_{\mathrm{a}}\mathbf{H}_{\mathrm{a}}^{\mathrm{T}}$$

$$\mathbf{G}_{\mathrm{HWE}} = [2\sum_{j=1}^{m} p_j(1-p_j)]^{-1}\mathbf{H}_{\mathrm{a}}\mathbf{H}_{\mathrm{a}}^{\mathrm{T}}$$

For HWE the $h_a[i, j]$ coefficients are commonly used and are given in [1] (un-numbered equation on page number 1299) as:

Genotype	Coefficient	
AA	$2-2p_j$	
Aa	$1-2p_j$	[S2.1]
aa	$-2p_j$	

Note that in the text of [1], $p_j = p_A$ and for comparison we continue to use both subscript *j* and subscript *A* as in [1].

For NOIA the $h_a[i, j]$ coefficients are given in [1] in their Equation 1 as:

Genotype	Coefficient	
AA	$-(-p_{Aa}-2p_{aa})$	
Aa	$-(1-p_{Aa}-2p_{aa})$	[S2.2]
aa	$-(2-p_{Aa}-2p_{aa})$	

These are equivalent forms. The allele frequencies can also be calculated from the genotype frequencies by: $p_j = p_A = p_{AA} + \frac{1}{2} p_{Aa}$ and $p_a = 1 - p_j = p_{aa} + \frac{1}{2} p_{Aa}$. Substituting these into S2.2 gives S2.1 as shown below.

Genotype	Coefficient
AA	$-(-p_{Aa}-2p_{aa}) = p_{Aa}+2p_{aa} \quad = 2p_a \quad = 2(1-p_j) \quad = 2-2p_j$
Aa	$-(1-p_{Aa}-2p_{aa}) = p_{Aa}+2p_{aa}-1 \quad = 2p_a-1 \quad = 2(1-p_j)-1 \quad = 1-2p_j$
aa	$-(2-p_{Aa}-2p_{aa}) = p_{Aa}+2p_{aa}-2 = 2p_a-2 \quad = 2(1-p_j)-2 \quad = -2p_j$

Example. Let $p_{aa} = 0.3$, $p_{Aa} = 0.2$ and $p_{AA} = 0.5$; so $p_A = p_j = 0.6$ and $p_a = 0.4$. The locus is not in HWE as $2p_A p_a = 0.48 \neq p_{Aa}$. Then the coefficients are as follows:

Genotype	From S2.1		From S2.2	
AA	2 – 2 x 0.6	= 0.8	-(-0.2 – 2 x 0.3)	= 0.8
Aa	1 – 2 x 0.6	= -0.2	-(1 – 0.2 – 2 x 0.3)	= -0.2
aa	– 2 x 0.6	= -1.2	-(2 – 0.2 – 2 x 0.3)	= -1.2

In conclusion the difference between $\mathbf{G}_{HWE}$ and $\mathbf{G}_{NOIA}$ lies only in their scaling and not in the contrasts used to calculate the elements of $\mathbf{H}_a$.

Literature Cited

1. Vitezica ZG, Legarra A, Toro MA, Varona L. Orthogonal Estimates of Variances for Additive, Dominance, and Epistatic Effects in Populations. Genetics. 2017;206:1297–307.
