# Supplementary material for: Genomic dissection of maternal, additive and non-additive genetic effects for growth and carcass traits in Nile tilapia
Source: Genet Sel Evol. 2020 Jan 15;52:1. doi: 10.1186/s12711-019-0522-2 (PMC6964056; doi:10.1186/s12711-019-0522-2)
Supplement: Supplementary file 4 — Additional file 4. Assumptions on the nature of non-additive genetic variance and the impact on estimates of additive genetic variance. The file presents the numerical proof that the estimate of additive genetic variance is reduced when the non-additive variation is assumed to be additive-by-additive epistasis rather than dominance, and this reduction is of the order of 3/8 epistatic variance. [file 12711_2019_522_MOESM4_ESM.pdf]

Additional file 4: Assumptions on the nature of non-additive genetic variance and the impact on estimates of additive genetic variance

Genomic dissection of maternal, additive and non-additive genetic effects for growth and carcass traits in Nile tilapia

R Joshi, THE Meuwissen, JA Woolliams and HM Gjøen

Joshi et al. (2018)[1] showed how the variance components of the basic factorial mating design used in this study were translated into estimates of maternal, dominance and additive variances related to a pedigree. The basic factorial mating design had 3 core components (V_{Sire}, V_{Dam}, and V_{Fsib}) which can be related to the covariances (C) between individuals, *i* and *j*, assuming a mean is fitted to the population.

i, *j* no common parent (U), $C_U = 0$ (1)

i, *j* paternal half-sibs (PHS), $C_{PHS} = V_{Sire}$ (2)

i, *j* maternal half-sibs (MHS), $C_{MHS} = V_{Dam}$ (3)

i, *j* full-sibs (FS), $C_{FS} = V_{Sire} + V_{Dam} + V_{Fsib}$ (4)

For this population *i* and *j* were in generation 22, and Joshi et al. (2018)[1] published the main results with a base set at generation 20.

Dominance. Assuming the non-additive genetic variation was primarily arising from dominance then Joshi et al. (2018)[1] showed:

$$C_U = (4\sigma^2_A + \sigma^2_D)/16 \quad (5)$$

$$C_{PHS} = (6\sigma^2_A + 2\sigma^2_D)/16 \quad (6)$$

$$C_{MHS} = (6\sigma^2_A + 2\sigma^2_D)/16 + \sigma^2_M \quad (7)$$

$$C_{FS} = (8\sigma^2_A + 4\sigma^2_D)/16 + \sigma^2_M \quad (8)$$

The fitted mean will account for the genotypic drift from the base generation, which is represented by C_U, and Equation 5 can be subtracted from the (6), (7) and (8).

$$C_{PHS} = (2\sigma^2_A + \sigma^2_D)/16 \quad (9)$$

$$C_{MHS} = (2\sigma^2_A + \sigma^2_D)/16 + \sigma^2_M \quad (10)$$

$$C_{FS} = (4\sigma^2_A + 3\sigma^2_D)/16 + \sigma^2_M \quad (11)$$

Solving these equations and equating them to (2) to (4) results in the following:

---

[1] Joshi R, Woolliams JA, Meuwissen THE, Gjøen HM. Maternal, dominance and additive genetic effects in Nile tilapia; influence on growth, fillet yield and body size traits. Heredity (Edinb). 2018;120:452-62.

- σ^2_M is estimated as $C_{MHS} – C_{PHS}$.
- σ^2_D is estimated as $16(C_{FS}–C_{PHS}–C_{MHS}) = 16V_{Fsib}$
- σ^2_A is estimated as $16C_{PHS}-8(C_{FS}-C_{MHS}) = 8(V_{Sire} - V_{Fsib})$.

Epistasis[2]. Consider now an assumption that the non-additive genetic variation was primarily arising from A#A, denoted σ^2_I :

$$C_U = (16\sigma^2_A + 4\sigma^2_I)/64 \quad (5)$$

$$C_{PHS} = (24\sigma^2_A + 9\sigma^2_I)/64 \quad (6)$$

$$C_{MHS} = (24\sigma^2_A + 9\sigma^2_I)/64 + \sigma^2_M \quad (7)$$

$$C_{FS} = (32\sigma^2_A + 16\sigma^2_I)/64 + \sigma^2_M \quad (8)$$

As with dominance the fitted mean removes C_U and this is subtracted from remaining covariances.

$$C_{PHS} = (8\sigma^2_A + 5\sigma^2_I)/64 \quad (6)$$

$$C_{MHS} = (8\sigma^2_A + 5\sigma^2_I)/64 + \sigma^2_M \quad (7)$$

$$C_{FS} = (16\sigma^2_A + 12\sigma^2_I)/64 + \sigma^2_M \quad (8)$$

The solutions to these equations are:

- σ^2_M is estimated as $C_{MHS} – C_{PHS}$.
- σ^2_I is estimated as $32(C_{FS}–C_{PHS}–C_{MHS}) = 32V_{Fsib}$
- σ^2_A is estimated as $28C_{PHS}-20(C_{FS}-C_{MHS}) = 8V_{Sire} - 20V_{Fsib} = 8(V_{Sire} - V_{Fsib}) – 12V_{Fsib}$.

Therefore the estimate of σ^2_A from this design is reduced when the non-additive variation is assumed to be additive-by-additive epistasis rather than dominance, and this reduction is of the order of 3/8 σ^2_I.

---

[2] Cockerham CC. An extension of the concept of partitioning hereditary variance for analysis of covariances among relatives when epistasis is present. *Genetics.* Genetics Society of America; 1954;39(6):859-82.
