# Supplementary material for: Genomic dissection of maternal, additive and non-additive genetic effects for growth and carcass traits in Nile tilapia
Source: Genet Sel Evol. 2020 Jan 15;52:1. doi: 10.1186/s12711-019-0522-2 (PMC6964056; doi:10.1186/s12711-019-0522-2)
Supplement: Supplementary file 5 — Additional file 5. Impact of inbreeding depression on models. The file contains the tables with the models without individual homozygosity as the covariate to account for the impact of the inbreeding depression in the models. [file 12711_2019_522_MOESM5_ESM.pdf]

Additional file 5: Impact of inbreeding depression in the models

Genomic dissection of maternal, additive and non-additive genetic effects for growth and carcass traits in Nile tilapia

R Joshi, THE Meuwissen, JA Woolliams and HM Gjøen

Both the models with HWE and NOIA approaches were fitted without individual homozygosity as the covariate to account for the impact of the inbreeding depression in the models. The summaries of the variance parameters are presented in the tables below, which are different than the variance parameters presented in Table 3.

Table S5.1: Heritabilities, ratio and phenotypic variance, for the models of best fit for different traits. The relationship matrices were constructed with HWE approach. Models were not fitted with individual homozygosity as the covariate.

HWE approach - Without individual homozygosity											
**Traits**	**Model**	**h^2**	**se**	**e_{aa}^2**	**se**	**H^2**	**se**	**m^2**	**se**	**σ_P^2**	**se**
**BD**	AME	0.17	0.05	0.19	0.11	0.36	0.10	0.08	0.05	0.58	0.04
**BWH**	AME	0.11	0.04	0.22	0.11	0.33	0.10	0.08	0.05	7540	548
**BL**	AM	0.10	0.03					0.08	0.05	3.41	0.22
**FW**	AM	0.11	0.04					0.08	0.05	1252	82
**BT**	A	0.20	0.04							9.96	0.50
**FY**	A	0.21	0.04							9.45	0.47

Table S5.2: Heritabilities, ratio and phenotypic variance, for the models of best fit for different traits. The relationship matrices were constructed with NOIA approach. Models were not fitted with individual homozygosity as the covariate.

NOIA approach - Without individual homozygosity											
**Traits**	**Model**	**h^2**	**se**	**e_{aa}^2**	**se**	**H^2**	**se**	**m^2**	**se**	**σ_P^2**	**se**
**BD**	AME	0.16	0.04	0.16	0.09	0.32	0.09	0.08	0.05	0.54	0.04
**BWH**	AME	0.10	0.04	0.19	0.10	0.29	0.09	0.09	0.05	7110	499
**BL**	AM	0.09	0.03					0.08	0.05	3.38	0.22
**FW**	AM	0.10	0.03					0.08	0.05	1236	80
**BT**	A	0.18	0.04							9.74	0.46
**FY**	A	0.19	0.04							9.23	0.44

Table S5.3: Literature review for inbreeding depression in some species of aquaculture. The inbreeding depression is expressed as the percentage decrease in the trait value per 10% increase in the inbreeding coefficient.

Species	Trait	Inbreeding depression
Atlantic salmon [1]	BW	-0.6 to -2.6%
Rainbow trout [2]	BW	-2.3%
Rainbow trout [3]	BWH	-1.6 to -5.0%
Coho salmon [4]	BWH	-1.5% to -1.7%

BW- Body Weight, BWH- Body Weight at Harvest

Literature cited

1. Rye M, Mao ILL. Nonadditive genetic effects and inbreeding depression for body weight in Atlantic salmon (Salmo salar L.). Livest Prod Sci [Internet]. Elsevier; 1998 [cited 2018 Sep 4];57:15–22. Available from: https://www.sciencedirect.com/science/article/pii/S0301622698001651

2. Hu G, Wang C, Da Y. Genomic heritability estimation for the early life-history transition related to propensity to migrate in wild rainbow and steelhead trout populations. Ecol Evol [Internet]. 2014 [cited 2015 Oct 16];4:1381–8. Available from: http://doi.wiley.com/10.1002/ece3.1038

3. Pante MJR, Gjerde B, McMillan I. Effect of inbreeding on body weight at harvest in rainbow trout, Oncorhynchus mykiss. Aquaculture [Internet]. Elsevier; 2001 [cited 2018 Sep 4];192:201–11. Available from: https://www.sciencedirect.com/science/article/pii/S0044848600004671

4. Neira R, Díaz NF, Gall GAE, Gallardo JA, Lhorente JP, Manterola R. Genetic improvement in Coho salmon (Oncorhynchus kisutch). I: Selection response and inbreeding depression on harvest weight. Aquaculture [Internet]. Elsevier; 2006 [cited 2018 Sep 4];257:9–17. Available from: https://www.sciencedirect.com/science/article/pii/S0044848606001839
